# Influence of critical care nurse education and work environment on outcomes of mechanically ventilated older adults

**DOI:** 10.1186/cc12442

**Published:** 2013-03-19

**Authors:** DM Kelly, LH Aiken

**Affiliations:** 1 University of Pittsburgh, PA, USA; 2 University of Pennsylvania, Philadelphia, PA, USA

## Introduction

Demand for critical care services is increasing yet a comprehensive understanding of how critical care nurses - the largest group of ICU direct care providers - impact outcomes remains unclear. The purpose of this study was to determine how critical care nurse education (hospital proportion of bachelor's prepared ICU nurses) and ICU work environment influenced 30-day mortality of mechanically ventilated older adults.

## Methods

A multi-state cross-sectional nurse survey was linked to hospital administrative data and Medicare claims (2006 to 2008). The final sample included 55,159 mechanically ventilated older adults in 303 hospitals. Logistic regression modeling was employed to jointly assess the relationship of critical care nurse education, work environment and staffing on 30-day mortality while adjusting for hospital and patient characteristics and accounting for clustering.

## Results

A 10% increase in the proportion of ICU nurses with a bachelor's degree or higher was associated with 2% lower odds of death while controlling for patient and hospital characteristics. Patients cared for in better work environments experienced 11% lower odds of risk-adjusted death than those cared for in poorer ICU work environments.

## Conclusion

Patients cared for in hospitals with a greater proportion of bachelor's prepared ICU nurses and in better ICU work environments experienced significantly lower odds of death. As the demand for critical care services increases, attention to the education level of ICU nurses and ICU work environment may be warranted to optimize currently available resources and potentially yield better outcomes.

